# Child protection, domestic violence, and ethnic minorities: Narrative results from a mixed methods study in Australia

**DOI:** 10.1371/journal.pone.0226031

**Published:** 2019-12-04

**Authors:** Pooja Sawrikar

**Affiliations:** School of Human Services and Social Work (HSV), Griffith University (GU), Southport, Queensland, Australia; Chinese Academy of Medical Sciences and Peking Union Medical College, CHINA

## Abstract

**Objective:**

To help address a long-standing gap in research and knowledge, this paper explores the issue of what service providers need to be aware of to best meet the needs of ethnic minority children and families who have come to the attention of child protection authorities and have substantiated reports of domestic violence.

**Method:**

The results are written in narrative form, combining informed insider perspectives with a small subset of data drawn from a larger rigorous mixed methods study in Australia, that involved an exhaustive literature review, review of 120 randomly selected case files, and in-depth qualitative interviews with 29 ethnic minority families involved in the child protection system and 17 child protection caseworkers.

**Results and significance:**

Three issues for ethnic minorities relating to the nexus of child protection and domestic violence are identified: (i) being in the child protection system tarnishes *family name*, which is greatly valued, leading to a preference for child maltreatment and family violence to remain private, and for compliance with Apprehended Violence Orders (AVOs) and service uptake/engagement to be low, (ii) *family cohesion* is also highly valued, so family violence victims may sacrifice their own personal safety to protect the family unity and cultural safety of their children, and (iii) family violence interacts with cultural factors for ethnic minorities but does occur in all families; attributing it to race or culture would be *racism*. Several implications for practice are identified, falling under a broad umbrella approach that asks for child protection authorities and family violence agencies to work collaboratively. A call for empirically rigorous future research is also made to ensure practice is evidence-based.

## Introduction

### Background, aims, and significance

Little is known about the complex and niche nexus of child protection and domestic violence issues among ethnic minority communities in Western countries like Australia. This reflects a broader historical trend of low research attention being paid to ethnic minorities in Western child protection systems [1–7]. To help address this gap, the then New South Wales (NSW) Department of Community Services (DoCS) in Australia (now NSW Department of Family and Community Services; FaCS) funded a three-year Postdoctoral Fellowship (2007–2010) on Culturally and Linguistically Diverse (CALD) children and families in the NSW child protection system (CPS).

The aim of this paper is to report on the findings from that study that relate to domestic violence (DV) among ethnic minority families in the CPS. The main research question is: what do child protection and family violence service providers need to be aware of to best meet the needs of CALD children and families who have come to the attention of child protection authorities and have substantiated reports of DV? While the results are based in the state of NSW in Australia, they do have some transferability to other comparable Western countries such as the US, UK, Canada, and New Zealand. Thus, this is a significant contribution to the literature because it helps progress the field with identification of key themes, as well as summary points translated for practice.

### Defining the group clearly

CALD groups are differentiated from Indigenous Australians (Aboriginal and/or Torres Strait Islander) because they have a migration history and so are not First People. They are also differentiated from Anglo Australians (Saxon and/or Celtic) because they are non-mainstream in any of the four main dimensions of ethnicity — race, culture, language, and religion [8]. Thus, CALD groups usually come from non-English speaking backgrounds (NESBs), and countries higher on collectivism than Australia—those that see the family, as opposed to the individual, to be the basic unit of society [9, 10].

According to the seminal work of Hofstede (2001), the US, Australia, and UK have the highest ratings on ‘Individualism’ (IDV) of 91, 90, and 89, respectively; Western European countries rank next highest (e.g. Netherlands = 80, Denmark = 74, France = 71, Switzerland = 68); East European countries rank thereafter (e.g. Czech Republic = 58, Hungary = 55); and South American, Asian, African, and Middle Eastern countries rank lowest on IDV (e.g. India = 48, Japan = 46, Brazil = 38, Iraq = 38, Saudi Arabia = 38, Philippines = 32, Kenya = 27, China = 20, Singapore = 20, El Salvador = 19, Pakistan = 14, and Ecuador = 8).

Hofstede (2001) has also identified the ratings of countries on the ‘Power Distance Index’ (PDI), which he defines as measuring “the extent to which less powerful members of organisations and institutions (e.g. the family) accept and expect that power is distributed unequally, so it represents inequality and suggests that a society’s level of inequality is endorsed by the followers as much as by the leaders”. It is therefore not wholly a measure of traditional gender roles but does include them, and the data shows that there is an observable trend between PDI and collectivism. For example, scores on PDI for the US = 40, Australia = 36, UK = 35, Netherlands = 38, Denmark = 18, France = 68, Switzerland = 34, Czech Republic = 57, Hungary = 46, India = 77, Japan = 54, Brazil = 69, Iraq = 80, Saudi Arabia = 80, Philippines = 94, Kenya = 64, China = 80, Singapore = 74, El Salvador = 66, Pakistan = 55, and Ecuador = 78.

Since the term ‘CALD’ only draws attention to two of the four main dimensions of ethnicity (culture and language), it is not sufficient for naming all the barriers along which this group of people might face [11]. Thus, ‘ethnic minorities’ is the preferred term used here. All synonymous terms (‘ethnic minorities’, ‘CALD’, ‘NESB’) include refugees and asylum seekers, but their needs and experiences are unique to the wider category, so should not be seen as sufficient for understanding this group [12, 13].

The literature on acculturation shows that migrants (newly arrived, well-established, and across generations) do not assimilate because of a *continual* management of the need to adapt to the new culture against a competing need to preserve the one of origin [14]. People and groups tend to lean toward ‘cultural preservation’ rather than ‘cultural adaptation’ if they experience racism and discrimination and expectations to assimilate; something they cannot achieve anyway because, as has been argued in-depth elsewhere, “people cannot (pretend to) be something they are not” (p.58) [5]. That is, efforts to ‘fit in’ are capped by visible and other differences such as in skin colour, accents, and religious attire that then socially propel them out of the ‘in-group’ [5, 15].

One implication of these social forces is that cultural norms, values, and traditions from the country of origin — such as that of collectivism—remain pertinent to the daily lives of people from ethnic minority communities. In the context of child protection and domestic violence, collectivism has relevance because family needs, most especially its reputation, are likely to be prioritised over the needs of individuals [16]. It is for this reason that it plays a critical role in theoretically framing the needs and experiences of ethnic minorities.

Importantly, ‘individualism’ and ‘collectivism’ are broad heuristic categories, and therefore stereotypes, which people, families, and sub-groups do not always fit. Therefore, the terms are not being used here to ‘box’ ethnic minorities (and other groups), but instead to help provide a culturally meaningful and relevant ‘lens’ by which to interpret their needs and experiences, including variation within the group.

The other implication of these social forces is that since assimilation is not possible, it necessarily moves the dialogue toward support of and respect for cultural diversity. A third of Australia’s population is comprised of its ‘*ethnically different’*; first and second generation Australians originating from non-English speaking countries [17]. This degree of multiculturalism suggests that the need for cultural awareness has reached ‘critical mass’ and is not really optional knowledge; *all* relevant authorities and service providers should be aware of the key issues relating to the nexus of child protection, domestic violence, and ethnic minorities.

## Method

### Stage 1

The Postdoctoral Fellowship was an empirically rigorous mixed methods study, comprised of three stages. Stage 1 exhaustively reviewed the relevant academic literature and publicly available policy guidelines for the experiences, needs, and challenges of ethnic minority children and families in the CPS, and of CP staff working with them. Particular attention was paid to four CALD groups that NSW DoCS selected, based on anecdotal evidence of either over- or under-representation in the NSW CPS–Chinese, Lebanese, Pacific Islander, and Vietnamese. Practice resources, service delivery models and efficacious interventions were also scoped for. Themes drawn from the scholarly literature on domestic violence and ethnic minorities published between 2000–2016 has been included in this paper to ensure the relevant knowledge has been updated.

### Stage 2

In Stage 2, 120 case files across six cultural groups (20 per group) were randomly selected by the researcher from a deidentified list provided by NSW DoCS, and then reviewed against a comprehensive scoping template informed by the literature review: Chinese [CHN]; Lebanese [LEB]; Pacific Islander—Samoan and Tongan [PAC]; Vietnamese [VIE]; Aboriginal [ABR]; and Anglo [ANG]. The sample was representative of gender as 62/120 (51.7%) cases were male, and the age range was 1–19 years (rounded up; mean = 11.7 years).

Case files were exhaustively scoped for: reported types of abuse and neglect; reported risk factors of abuse and neglect; reported strengths of children and families; reported issues with the CPS by families; reported assistance provided to families; examples of culturally appropriate and inappropriate practice; and examples of personal, organisational, or institutional barriers to culturally appropriate practice.

### Stage 3

Of the 29 CALD family participants in Stage 3, which involved semi-structured interviews: 10 were male (34.5%); they varied in age from 22–67 years (mean = 42.2 years); and they came from a diverse range of ethnic backgrounds (Egypt, Iraq, Jordan, Lebanon, Turkey, Cambodia, Vietnam, Philippines, Sudan, Burundi, Ethiopia, Ghana, Sierra Leone, Greece, Macedonia, Serbia, Maori, Samoa, Argentina, and Netherlands). Of the 17 CP caseworker participants in Stage 3: only two were male (12%), however this is consistent with the typically female-dominated social-work-force; they ranged in age from 23–59 years (mean = 33.9 years); the 13 NESB caseworkers originated from Afghanistan, Egypt, Lebanon, Burma, Laos, Philippines, Vietnam, India, Uruguay, and Ghana (and four were born in Australia, so are second generation); and four interviewees were Anglo caseworkers all born in Australia. (Note: names have not been provided here to protect participants’ anonymity and confidentiality. All subjects gave their informed written consent for inclusion before they participated in the study. The study was conducted in accordance with the Declaration of Helsinki, and the protocol was approved by the Human Research Ethics Committee (HREC) of University of New South Wales (UNSW; Approval number 07215).

Interviews explored: norms, traditions, beliefs and practices that influence the way ethnic minority children are raised and family issues are addressed; reasons underpinning the entry of ethnic minorities into the CPS; services provided to ethnic minorities; perceived commonalities and differences in working with ethnic minority, Aboriginal, and Anglo children and families; how caseworkers take into account different cultural contexts for children, while adhering to one Australian child protection law; whether, and why, ethnic minorities and caseworkers prefer an ethnic-match; examples of effective and ineffective practice with interpreters; examples of culturally appropriate and inappropriate practice; the extent to which caseworkers perceive personal, organisational, and/or institutional barriers impeding culturally appropriate service delivery for ethnic minorities; the extent to which the CPS is perceived to sufficiently address the cultural needs of ethnic minorities; and suggestions for overcoming barriers to, and improving, the cultural appropriateness of child protection service delivery for ethnic minorities. Thus, interviews were in-depth and exhausted all themes relevant to the Fellowship.

### Methodological strengths and weaknesses and implications for future research

While a total sample of 120 case files is large and robust for identifying relevant themes, the sample size of 20 per cultural group is not sufficient for conducting statistical analyses to test for cross-cultural differences. Thus, caution should be exercised when making inferences about each of the six *populations* explored. Having said that, the qualitative interviews (Stage 3) did reach thematic saturation, and triangulating three different sources of data (literature reviews, case file reviews, and qualitative interviews), add to the methodological strengths of this study. That is, a mixed methods approach to theory, data collection, and data analysis all help improve the representativeness of a study’s findings. Thus, the results overall are representative of ethnic minorities and child protection staff who work with them.

Only data relating to domestic violence has been reported on here to address the research question (the results of the larger study are published elsewhere; [5]), but as a result this data cannot be said to be representative. The Stage 3 interviews did not focus on this specific issue to be able to provide in-depth exploration and data, leading to greater unweighted leanings on the Stage 1 literature review and Stage 2 case files. Information about all three stages has only been provided so that readers are able to gauge the rigour of the larger study and contextualise the relevant data from which it has been drawn. Under-reporting of DV in ethnic minority communities due to issues around family name, family cohesion, and racism (the three main themes discussed in the Results section) also impact on the ability of studies in this area to claim representativeness.

Thus, these limitations mean that further research in the area is essential. At this nascent stage of knowledge-building in a critically under-developed area, this paper does not claim to offer evidence-based principles of best practice but rather only ‘issues to consider’ and ‘suggestions for practice’. The paper takes a narrative form that draws on twenty years of research on ethnic minority affairs in relation to the issues of racism, sexism, culture, migration, mental health, domestic violence, and child protection, as well as the insider perspective of an ethnic minority person thereby additionally valuing emic methodologies. Overall, suggestions offer a starting place about what might constitute as good practice, but studies with more empirically rigorous methodologies are required in the future to test them.

## Results

### Cross-cultural prevalence of family violence and child maltreatment

Children who enter the CPS due to substantiated reports of maltreatment—physical abuse, sexual abuse, emotional abuse, inadequate supervision, and/or chronic neglect—may also have reports of DV, which has been acting as a risk factor to child maltreatment. Thus, multiple child safety issues might need to be addressed. When CP workers refer these families to DV (counselling and refuge) services, the service is likely to receive families with complex needs; compromises to child safety in the form of emotional abuse due to witnessing DV may be but one issue in need of address. As one example of such emotional abuse, a case file report said: *“Children exposed to ongoing and extensive DV*. *Witnessing NF (natural father’s) continual threats to kill NM (natural mother) and children” [ANG_case file]*.

Indeed, one key finding from Stage 2 was that not including financial issues/poverty, DV was the most common risk factor of child maltreatment (especially emotional abuse) in all six (and maybe all) cultural groups in Australia (see Tables 1 and 2). Specifically, DV was reported in between 47.4% and 80% of case files per cultural group. The results therefore show that prevalence of DV among families who have entered the CPS is high, along with ensuing referrals to specialist services in family violence.

**Table 1:** Reported generalist risk factors of child maltreatment by cultural group.

	N	DV (%)	AOD^a^ (%)	MH^b^ issues in carer (%)	Housing issues^c^ (%)
**Chinese**	19^d^	**9 (47.4)**	2 (10.5)	6 (31.6)	1 (5.3)
**Lebanese**	19^e^	**12 (63.2)**	6 (31.3)	9 (47.4)	6 (31.6)
**Pacific Islander**	16^f^	**11 (68.8)**	6 (37.5)	6 (37.5)	4 (25)
**Vietnamese**	20	**11 (55)**	6 (30)	6 (30)	1 (5)
**Aboriginal**	20	**16 (80)**	16 (80)	7 (35)	4 (15)
**Anglo**	20	**13 (65)**	12 (60)	8 (40)	3 (15)
**Total**	**114**	**72 (63.2)**	**48 (42.1)**	**42 (36.8)**	**19 (16.7)**

a–Alcohol and other drug issues.

b–Mental health.

c–Current, fear, or threat of homelessness.

d–One case excluded due to insufficient information.

e–Two cases were siblings in one family, so data is on 19 *different* case files to account for intra-familial co-variation.

f–Three cases were siblings in one family and another three were siblings in another family. Thus, the 2 x 2 additional children in each family (*n* = 4) not included to account for intra-familial co-variation.

**Table 2:** Reported types of child maltreatment by cultural group within DV cases.

	Reported types of child maltreatment					Total DV reports
	Emotional abuse	Neglect^a^	Physical abuse	Inadequate supervision	Sexual abuse	
**Chinese**	9	0	6	3	2	**9**
**Lebanese**	9	11	9	3	0	**12**
**Pacific Islander**	7	5	6	5	3	**11**
**Vietnamese**	9	6	7	5	2	**11**
**Aboriginal**	11	15	9	13	8	**16**
**Anglo**	12	13	10	5	7	**13**
**Total**	**57**	**50**	**47**	**34**	**22**	**72**

a–Neglect of basic needs and education.

However, the core business of CP workers is child safety, whereas the core business of DV service workers is family safety which includes victimised adults—mostly female, but no more qualitatively (as opposed to quantitatively) important to protect. As one reported example of DV among men: “*She hits me in front of the children*. *I told her not to do it*. *I try restraining myself*. *We men suffer*, *(we) can’t say anything” [CHN_case file]*. Of course, they are not independent goals, but differences in the focal point mean that a mutually beneficial exchange between agencies is in the best interests of the child and family.

Importantly, DV is a generalist issue; the physical, emotional, and sexual violence ‘looks the same’ and all families require equal attention in this regard. Having said that, DV interacts with cultural and other factors for ethnic minorities. The following section discusses these factors.

### Three key and inter-related themes

#### Family name

Various recent nationally representative data sources [18–20], indicate that one in six Australian women have experienced physical or sexual violence from a current or former partner since the age of 15, one in four women have experienced emotional abuse, one in two Australian men have experienced physical abuse, one in seven men have experienced emotional abuse, and at least one Australian woman a week is killed by a current or former partner. These high prevalence rates suggest that even though there have been significant increases in public awareness of DV since the 1990s [21], with more recent consolidated attention offered by DV campaigner Rosie Batty and the development of the *National Plan to Reduce Violence against Women and their Children 2010–2022* [22], DV still has a tendency to be seen as a private family matter. That is, there seems to be a culture to not intervene in “private” family affairs unless it is so extreme that it becomes noticeable, leading to a large threshold of silence and permissiveness.

Thus, cultural change is rarely swift, and as such, not all cases are reaching the attention of authorities such as the police and child protection systems. This means that the true prevalence of DV is still unknown [23, 24]. Nevertheless, some gauge of its prevalence in the community is offered (with national surveys as previously mentioned, and) when children enter the CPS with substantiated reports of maltreatment and DV, such as the data in Table 1. Critically, it shows that at least for cases that do reach the attention of authorities, prevalence is high for all groups (even if not to the same extent).

While the numbers indicate that DV is an issue in all families, this does not mean that it is qualitatively experienced the same across cultural groups. Specifically, the trend toward keeping DV a private family matter is intensified in collectivist communities, which ethnic minorities tend to be, because of a definitive value for family and therefore family reputation. As one example of this, a case file report said: “*NF indicated he did not normally discuss the family’s problems as this [DV] was a private matter” [CHN_case file]*.

In other words, the family name must be protected at all costs, including the safety of adult and child victims, whose individual needs for safety are seen as second to those of the familial or collective group. Coming to the attention of police and CP authorities is perceived as a great shame because it tarnishes family name and community standing, and in turn, risks social rejection and isolation from their ethnic community. Community acceptance is particularly critical to protect for ethnic minorities because it provides cultural safety in addition to sense of belonging (also see discussion under ‘Racism’ below). Thus, there is a preference for authorities to not ‘intrude’ in private family matters. As examples, two case file reports said: “*Mother refused to provide a statement to police and get photos taken of her injuries” [LEB_case file]*, and *“NM holds fears for her safety due to previous physical abuse by her husband that she was afraid to report as it would bring shame to the family” [LEB_case file]*.

On the other hand, state intervention represents authoritative protection from the perpetrator, and as such, may also be welcome by victims. However, their presence may only be welcome to the extent that it offers a ‘shake up’ or ‘threat’ to the perpetrator that family matters could escalate into legal ones, and thus provide short-term safety and respite from harm. Beyond that, the need to protect the family name remains over-arching, and more prolonged state intervention such as in the form of removal of children, may not be perceived as beneficial by the victims.

#### Family cohesion

As an example of ethnic minorities’ feelings of intrusion by authorities—whom ethnic minorities also tend to fear [25] — victims may feel coerced to take out an Apprehended Violence Order (AVO) on their perpetrator spouse, in which case they are unlikely to comply with it fully because the action or initiative would not be self-determined. While victims may welcome having a legal document that gives them the right to protect their own safety, it also represents a value for ‘self’ which is not socially rewarded in collectivist groups; keeping a perpetrator away from the family is seen as threatening the family unit and its cohesion or togetherness, paramount in collectivist cultures. Indeed, Rees and Pease (2007) report that in a sample of refugees in Australia, awareness of the legal ramifications for DV reduced its risk for some, but if victims did seek help they were considered “betrayers of their own culture” (p. 10) [26]. Evidence of the need to protect family cohesion was also found in the case files: “*Pressure from family to drop assault charges and AVO against father” [VIE_case file]*; *“An AVO in place*, *though father has indicated he will lie for the mother to keep the family together” [CHN_case file]*; and *“Mother taken out AVO on father but does not abide by Family Law Court (FLC) order—mother allows father to have contact during dinner time with the children” [LEB_case file]*.

The need to protect family cohesion is a particular burden on ethnic minority mothers. Collectivist communities tend to have traditional gender roles, with overtly patriarchal social structures; daily life is lived with explicit understanding that males have more power than females [5]. Several authors note that intimate partner violence (IPV) is higher in societies with overt traditional gender roles [27, 28]. Patriarchal gender roles also occur in individualistic countries (e.g. inequitable housework shares, inequitable divisions in full-time/part-time employment among families with children, inequitable access to promotions and management positions, etc.), but social forces strive to make gender imbalances more equal, leading to discourse or rhetoric that does not match the lived experience, but at the same time less traditional too [29]. That is, women in individualistic cultures still experience gender inequality despite overt and pervasive efforts to reduce it, because gender roles are only less traditional not *un*traditional.

Ethnic minority mothers who are victims of DV may be at odds with authorities in their construals of child safety. Authorities may perceive that an AVO offers child protection but mothers may perceive that children in a family unit that is not cohesive are subject to social rejection and isolation from their ethnic community and are therefore not protected. These mothers may sacrifice their own on-going ‘personal safety’ to protect the ‘cultural safety’ of their children; perceived as more important [30]. One way they attempt to resolve the dissonance between all their conflicting needs is by minimising the threat to their child’s personal safety. As two examples of this, case files said: *“AVO protecting mother and children from father*, *[but] mother not being protective*, *not enforcing AVO” [LEB_case file]*; and *“Mother not accepting any responsibility for keeping the children safe*. *She has indicated she will leave the father in the event of another domestic incident*, *however she has said this in the past and not followed through” [LEB_case file]*.

Thus, ‘failing to protect children’ can mean different things to a CP caseworker and an ethnic minority woman victim of DV. Arguably, if ethnic minority women are given full acknowledgment of just how difficult and indefensible their predicament is, they may then be able to perceive risk to their child’s personal safety more accurately, and in turn, develop a sense of empowerment and agency needed to leave the harmful situation and accept the loss of cultural safety. In other words, empathy and value for the protective factors that collectivism and cultural preservation offer — cultural safety, family cohesion, and community belonging — may help her perceive the threat of safety to her children in the way the caseworker perceives it, rather than downplaying or denying it. One example of successful engagement was found in the case files: *“NM getting calls from mother and father-in-law saying ‘we’ll fix it between us’*. *NM said ‘no it’s too late*, *I’m protecting my daughter’*. *NM saw the counsellor who made her realise that she needs to protect her children” [LEB_case file]*. Full and genuine empathy may also help counteract any fears the mother is having that the caseworker has come with ‘Western beliefs’ about choice and freedom and independence that she may not have (perceived or actual). As an example, Adames and Campbell (2005) say, “the acculturative process and new economic demands may involve shifts in (standard) gender roles and power relations … Reluctant to accept these, (Latino men in the US may) resort to other means of asserting themselves such as IPV. Thus, exposure to mainstream U.S. culture and more egalitarian values and attitudes … could be more harmful than liberating” (p. 1342–1343) [31].

Most importantly, demonstrating this empathy is an example of cultural competency. It meets the needs of ethnic minority children and families in ways that *could* lead to better protection from harm, rather than imposing expectations on her she may not be able to meet and then risk judging her for failing to do so [32].

As it is, “parents who are the main support for children in providing nurturance and protection may not be able to do so when they are exposed to, or are victims of, (domestic) violence themselves” (p. 162) [33]. Thus, victims of DV from all cultural backgrounds find it difficult to leave for a range of psychological reasons, such as through the (battered) effects of “depression and lowered self-esteem” (p. 1353) [31] or self-blame [34].

There may also be fatalistic beliefs such as “accepting it is your lot … and a burden you have to carry for the rest of your life” (p. 1357) [31], or beliefs about marital permanence [24, 16], which may or may not be related to culture or religion. Victims may additionally fear leaving their husband “because they’re already used to him (‘better the devil you know’) … and are afraid to face life alone, more so if children are involved” (p. 1358) [31]. There could be financial dependency on husbands, causing them to stay in abusive relationships for longer [35], and for minorities this may also be tied in with fear of deportation if their visas depend on their perpetrator spouse [36] and/or low English proficiency which may affect their opportunity to work [37]. (Note: the ‘family violence exception’ of the *Migration Act 1958* means that victims do not have to stay in an abusive relationship for two years before being eligible to apply for permanent residency. However, this comes with other challenges for victims in terms of providing evidence of the abuse [38], making legal assistance critical). Finally, lack of supportive and extensive family networks to help with child-rearing—typically abundant in the country of origin because of the communal role of families, but then lost after migration—may make it hard for the woman victim to leave, who feels she has no ‘family capital’ to help her navigate a future independent of her perpetrator spouse.

If CP caseworkers and DV service providers fail to understand, acknowledge, document, and/or attempt to meet this deeply entrenched conflict for ethnic minority women DV victims—for both the personal and cultural factors that would need to be taken into account for them—it would be an example of poor practice. Indeed, “a dialectical relationship exists between humans and environment, (so) individuals have the ability to cope, resist, mitigate, envision new possibilities and change their life circumstances through interpreting the meaning of their experiences, historical problems of domination, alienation, and social struggle” (p. 614) [29], but these processes—which allow a woman victim to “make decisions (she) may not have made otherwise” (p. 591) [39]—take time.

Fear of the perpetrating spouse for disclosing abuse to authorities may also prevent victims of DV from seeking professional help. As Giglio (1997) says, “in cultures where men are considered superior to women, they may fear reprimand if they tell of abuse at home” (p. 5) [40]. Arguably, women victims may only agree to stay in a DV refuge if they perceive their or their children’s lives are at risk; the literature does report higher thresholds of frequency or severity of IPV among several minority groups before victims consider seeking help [31, 41–43]. Importantly, fear of disclosing abuse to authorities and service providers is not exclusive to ethnic minority families (58% of women who completed the 2012 Personal Safety Survey never contacted police [18] and 24% never sought advice or support; ANROWS 2012 [19]), but it may be heightened in families with overt traditional gender roles. As one example of this from the Stage 3 interviews, one male participant said: “*In Iraq*, *the famous point in our culture is — they don’t go [to] the police*, *because it’s a shame for the woman [to] bring the police for the husband” [CALD family interviewee]*.

#### Racism

It is reported in the literature that social service uptake and engagement tend to be lower from ethnic minority communities [44]. This is primarily due to normative reliance on their own ethnic community for help and support regarding family problems [45]. It also reflects the need to keep family matters private and avoid social stigma [46]. As an example, one case file report said: “*NF indicating he wants to keep his family together*. *After some discussion*, *NF agreed to supports/referrals*, *originally hesitant due to shame/embarrassment*. *NF*: *‘Can we keep confidential*, *can spark more problems*. *Wouldn’t want matter exposed’” [CHN_case file]*.

However, there are also system failures that compound this trend. For example, interpreters may not be provided to families low in English proficiency, who then remain unaware of the full implications of the involvement of CP agencies in their families [5]. Alternatively, the provision of an interpreter may be seen as sufficient for having met their needs, meaning that cultural, racial, and religious factors remain unmet.

More significantly, ethnic minority communities may not engage with authorities or the service sector because of fears of racism. As Kanuha (1994) puts it [47], “because of the power structure of many Western legal and social institutions as Eurocentric and androcentric, many ethnic minority women do not want to bring attention to their problems for fear of stigmatising their family and communities” (p. 279, cited in [48]).

There are real grounds for these feelings. For example, DV refuges may not provide culturally sensitive services, which victims fear [49] or expect [16], and could be heightened in ‘anti-immigration climates’ [36]. The literature also identifies racism among police—a typically male- and white-dominated organisation—which can make training about racism for minority women victims of DV challenging [50].

Additionally, some ethnic minority families and communities in Australia may perceive the removal of their children as a repeat of the ‘Stolen Generations’ and the Northern Territory [NT] ‘intervention’; that the need for child safety is assumed to be greater in non-mainstream communities and therefore becomes racially targeted. In specific cases, such a response may be a defensive one to deflect from the violence occurring in their family, but unfortunately it does not negate the validity of the threat, and perception of threat, that cultural safety is at risk.

In this regard, Weston-Scheuber’s (2007) article is seen as a ‘must read’ for practitioners in the field [51]. She discusses mainstream Anglo-Celtic culture and how it too perpetuates gendered violence; that it is not something that simply belongs to societies with *overtly* traditional gender roles. Ahmad et al. (2009) say, “there is a strong need to improve women’s social status in the South Asian community (in Canada). It is imperative to educate the community about gender inequality and its harmful linkages to domestic stress and partner abuse with serious consequences not limited to the victims. To trigger critical self-reflections for a positive change, an emphasis on the trans-generational consequences of abuse could be particularly beneficial due to its collectivistic and familial cultural values” (p. 619) [29]. While this call is accurate, within an emic framework that values social constructionism, the call for gender status equality to minimise the transference of the effects of abuse into future generations is actually cross-cultural.

## Discussion

### Summary of implications for practice

Evidently, much is happening in the space that occupies the nexus of child protection, domestic violence, and ethnic minority children, families, and communities. Informing practitioners of how best to navigate this space is in the best interests of children and adults unsafe from harm. To this end, this article offers the following suggestions for knowledge and practice. It is intended that should staff from CP agencies and DV services be aware of these issues, it can aid in their exchange because knowledge is not siloed off between agencies. However, CP and DV staff would still need to take carriage for how each suggestion would be implemented and cross-checked within their organisational practices and policies.

All staff should:

- Appreciate that ethnic minority DV victims share personal/psychological barriers to leaving the harmful situation with Anglo and Indigenous DV victims, thereby paying attention to cross-cultural similarities.
- Appreciate that the pressure to keep DV private in ethnic minority/collectivist communities is driven by an *utmost* value for family name/reputation, thereby paying attention to cross-cultural differences.
- Appreciate that protecting the family name avoids social isolation and exclusion from their ethnic community, which may be seen as more important to protect than their child’s personal safety; empathy for this dilemma may help improve the DV victim’s accuracy of risk perception.
- Ensure that referrals for all types of presenting child maltreatment issues have been made/considered (e.g. specialist child sexual abuse services if relevant).
- Ensure that referrals with culturally appropriate DV refuges are made.
- Ensure that victims receive legal advice regarding the ‘family violence exception’ of the *Migration Act 1958*.
- Use a feminist framework when engaging with women victims of DV, so that gendered power inequality at the systemic/societal level is not omitted from service engagement with individual clients.
- Use a feminist framework appropriately when engaging with men victims of DV (e.g. mental health issues in the female perpetrator may also need to be considered, and these in turn may be the result of gendered oppression; protecting male victims without stigmatising such female perpetrators is critical).
- Appreciate that their presence in an ethnic minority family is likely to be met with conflicting feelings of being both welcome and unwelcome by child and adult victims of maltreatment and violence.
- Be aware that in ethnic minority families, compliance with AVOs may only be partial because it threatens family cohesion, which is seen to be more important in the long-term to protect than personal safety.
- Acknowledge that traditional gender roles occur in all groups in Australia — Anglo, Indigenous, and ethnic minority — but that they are overtly accepted in ethnic minority communities. This helps see the role of patriarchy in ethnic minority communities and the burden it imposes on women victims of DV, but it also avoids the risk of seeing it as *only* belonging to ethnic minority communities.
- Be aware that service uptake may be low because DV victims fear reprimand from the perpetrator. This is true of all families regardless of their cultural background, but may be intensified in ethnic minority groups. To help address this, confidentiality must be repeatedly assured to ethnic minority families, who also fear tarnishing the family name should it be known that service support was being accessed. This way, staff can assure families there is no reason to fear the community finding out about the abuse and therefore no justifiable reason for them to ‘suffer in silence’. It may also help to remind the DV victim that its occurrence is high in all cultures, which is why extensive supports in the local community are available, and which may help decrease any isolation or loneliness.
- Be aware that DV service uptake may be low because ethnic minority/collectivist communities typically rely on intra-familial rather than external support. However, services should also reflect on whether they are culturally competent to ensure low service uptake is not due to policies and practices that are not client-centred.
- Provide an interpreter if English proficiency is low, but not see this as sufficient for meeting the needs of ethnic minority families.
- Be aware that violence in non-mainstream families is at risk of being labelled a ‘cultural issue’, unlike in Anglo families, because of stereotyping. Qualitative differences in the experience of family violence across cultures should be understood as part of cultural awareness, knowledge, and sensitivity, not as evidence for racist attributions.

## Conclusion

Three inter-related issues for consideration with ethnic minority children and families in Australia are: (i) family name, (ii) family cohesion, and (iii) racism. These make the ‘cultural’ subjective experience of CP and DV unique for ethnic minority families, however the violence itself is cross-cultural. That is, DV is a generalist risk factor of child maltreatment in all groups. Thus, cross-cultural similarities must not be over- or under-emphasised, but rather given equal consideration to cross-cultural differences. The main implication of these three issues is that cultural and racial factors are not addressed when only an interpreter is provided; the needs of ethnic minority groups are more than just linguistic.

The suggestions for practice offered here should be evaluated for whether and how they translate to effective exchange between statutory and non-statutory agencies to best keep ethnic minority children and families safe from harm. This is particularly critical given that the prevalence of DV is high in all groups, but its true extent is higher than what the statistics reveal. Developing a solid and rigorous research body can aid in reducing the threshold of silence and permissiveness that currently surrounds DV in all groups.
